# CD44: A Multifunctional Cell Surface Adhesion Receptor Is a Regulator of Progression and Metastasis of Cancer Cells

**DOI:** 10.3389/fcell.2017.00018

**Published:** 2017-03-07

**Authors:** Linda T. Senbanjo, Meenakshi A. Chellaiah

**Affiliations:** Department of Oncology and Diagnostic Sciences, Dental School, University of MarylandBaltimore, MD, USA

**Keywords:** CD44, cancer, metastasis, hyaluronic acid, migration, angiogenesis, invasion, CD44-ICD

## Abstract

CD44 is a cell surface adhesion receptor that is highly expressed in many cancers and regulates metastasis via recruitment of CD44 to the cell surface. Its interaction with appropriate extracellular matrix ligands promotes the migration and invasion processes involved in metastases. It was originally identified as a receptor for hyaluronan or hyaluronic acid and later to several other ligands including, osteopontin (OPN), collagens, and matrix metalloproteinases. CD44 has also been identified as a marker for stem cells of several types. Beside standard CD44 (sCD44), variant (vCD44) isoforms of CD44 have been shown to be created by alternate splicing of the mRNA in several cancer. Addition of new exons into the extracellular domain near the transmembrane of sCD44 increases the tendency for expressing larger size vCD44 isoforms. Expression of certain vCD44 isoforms was linked with progression and metastasis of cancer cells as well as patient prognosis. The expression of CD44 isoforms can be correlated with tumor subtypes and be a marker of cancer stem cells. CD44 cleavage, shedding, and elevated levels of soluble CD44 in the serum of patients is a marker of tumor burden and metastasis in several cancers including colon and gastric cancer. Recent observations have shown that CD44 intracellular domain (CD44-ICD) is related to the metastatic potential of breast cancer cells. However, the underlying mechanisms need further elucidation.

## CD44 Introduction

**Figure 1:**
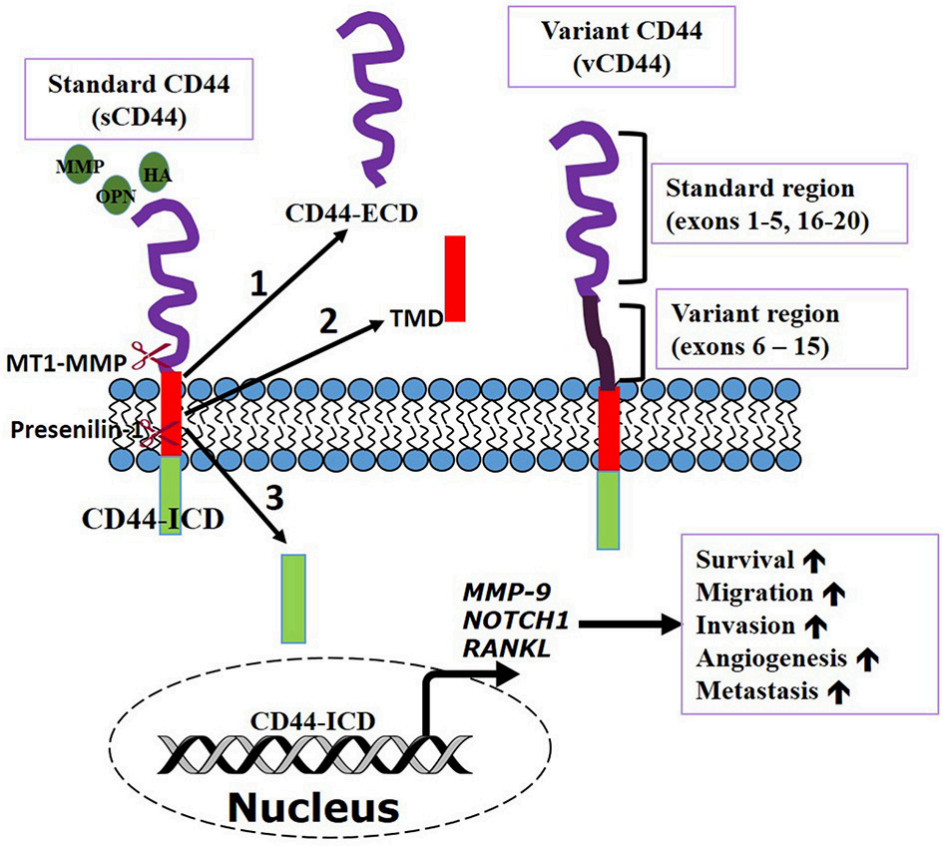
**CD44 transmembrane receptor function**. CD44, a multifunctional receptor can control biological functions involved in cancer cell dissemination and metastasis. CD44 can be sequentially cleaved by membrane type 1 matrix metalloprotease (MT1-MMP) and then presenilin-1/γ secretase induced by ligands [osteopontin (OPN), hyaluronic acid (HA), etc] binding. Cleavage produces **(1)** extracellular domain (ECD) fragment. **(2)** CD44β like peptide or transmembrane domain (TMD), and **(3)** CD44 intracellular domain (ICD) fragment. CD44—ICD translocates into the nucleus to activate transcription of genes important in metastasis and cell survival. Adapted from Thorne et al., 2004.

CD44 is a transmembrane glycoprotein also referred to as P-glycoprotein 1. It is encoded by a single gene on chromosome locus 11p13 (Underhill, 1992; Iczkowski, 2010). CD44 is ubiquitously expressed throughout the body and has a molecular weight of 85–200 kDa (Basakran, 2015). The standard CD44 (sCD44) is the conserved form with a molecular weight of about 85–90 kDa protein which is made of transcription of exons 1–5 and 16–20 that are spliced together (Rall and Rustgi, 1995; Rudzki and Jothy, 1997). The primary domains of CD44 are the extracellular domain (or ectodomain), the transmembrane domain, and the intracellular domain/cytoplasmic domain (Iczkowski, 2010). The extracellular domain interacts with the external microenvironment and senses stimuli in the external microenvironment (Underhill, 1992). The transmembrane domain provides an avenue for interacting with co-factors and adaptor proteins as well as directing lymphocyte homing (Underhill, 1992; Williams et al., 2013). CD44 intracellular domain (CD44-ICD) has a short-tail and long-tail configuration with functions in nuclear localization and transcription mediation (Okamoto et al., 2001; Williams et al., 2013). Our current understanding of the dual role of CD44 in cancer progression is summarized in Figure 1 below.

## Isoforms of CD44

Multiple isoforms of CD44 can be generated due to insertion of alternative exons at specific sites within the extracellular domain (Cichy and Puré, 2003). The variant isoforms of CD44 (CD44v) comprises of exon 6–15 spliced at various sites between exons 5 and 16 of the standard isoform (Goodison et al., 1999; Zeilstra et al., 2014). The expression of distinct CD44 isoforms appears to be necessary for the progression of human tumors (Günthert et al., 1995; Wang et al., 2009). One or multiple splice variants and standard CD44 may be expressed in cancer cells. There is an increased chance of expressing larger isoforms like that of CD44v8-10 in pancreatic cancers (Rall and Rustgi, 1995) and CD44v6 in colorectal cancer (Yamane et al., 1999). The expression of CD44v6 is well-known as a useful marker of tumor progression and prognosis in colorectal cancer (Yamane et al., 1999). In cultured supernatants from prostate cancer cell lines derived from bone metastasis (PC3), soluble CD44, and variant 6 isoform (v6) was identified, however, it was not identified in lymph node metastatic prostate cancer cell line (LNCaP, Stevens et al., 1996; Desai et al., 2009; Gupta et al., 2012). The switch from standard CD44 to CD44v6 improved survival and adhesion in prostate cancer (PC3) cells (Gupta et al., 2013b). The expression of CD44v6 in colorectal cancer is enhanced by cancer stem cells expression (Todaro et al., 2014).

## CD44 expression in normal and tumor cells

The ubiquitous transmembrane cell surface molecule CD44 is widely distributed in normal adult and fetal tissues. CD44 standard isoform was originally isolated from hematopoietic cells but now it is found in a variety of tissues e.g., central nervous system, lung, and epidermis. In comparison, the distribution of CD44 variant isoforms is restricted and expressed on a selection of epithelial cells (Sneath and Mangham, 1998). The isoforms with restricted distribution and exon sequence may have different functions as compared to the standard isoform of CD44. Keratinocytes, macrophages, and select epithelial cells express the variant CD44 (CD44v) isoforms and are present on tissues at various stages of development (Sneath and Mangham, 1998). In normal tissues, the importance of CD44 is vital in the regulation of hyaluronic metabolism, activation of lymphocytes, and release of cytokines. However, targeting of CD44 resulting in its loss leads to the disruption of hyaluronic metabolism, wound healing, and keratinocyte proliferation (Yu and Stamenkovic, 1999). Among many of CD44 functions, one is to make cell lines that are non-metastatic become more metastatic (Heider et al., 1993). The details that confer CD44 metastatic potential in human malignancies is the subject of further elucidation. Prostate cells that are benign express higher CD44 variant 5 isoforms (CD44v5), whereas neoplastic prostate cells express higher levels of CD44s (Dhir et al., 1997; Desai et al., 2009; Gupta et al., 2013a). Various breast cancer cells show abnormal expression of CD44 including heterogeneously expressing CD44 isoforms (Basakran, 2015).

## CD44 receptor-ligand interaction

CD44 is known to interact with various ligands and this interaction is crucial for its many cellular functions (Goodison et al., 1999). There are several well-known ligands of CD44 including hyaluronic acid (HA), osteopontin (OPN), collagens, and matrix metalloproteinases (MMPs) (Goodison et al., 1999). CD44 effects on cell migration and growth are dependent on its specificity to ligands (Weber et al., 1996).

### Hyaluronan (HA)

HA is a glycosaminoglycan that is a ubiquitous component of the extracellular membrane. It is considered the major ligand for CD44 and can bind CD44v isoforms that are ubiquitously expressed. Through binding of CD44, HA can activate cytoskeleton and matrix metalloproteinases (MMPs) signaling involved in tumor progression (Bourguignon et al., 2014). Multiple regions of the cytoplasmic domain of CD44 can promote enhancement of HA binding, however, the role of the cytoplasmic domain in mediating the binding does not require a specific amino acid sequence in T-lymphoma cells (Perschl et al., 1995). HA can exist in high molecular weight or low molecular weight form due to cleavage into varying sizes. In breast cancer cell lines, high molecular weight HA is involved in tumorigenesis, antiangiogenic and anti-inflammatory responses. However, low molecular weight HA has been shown to promote cell motility, CD44 cleavage and angiogenesis. Therefore, the size of HA ligand is important for the biological function (Louderbough and Schroeder, 2011).

### Osteopontin (OPN)

Several studies have demonstrated an elevated expression of OPN in highly invasive metastatic human cancers (Tuck et al., 2007). Integrin αvβ3 and CD44 are receptors for OPN and can interact with α_*v*_β_3_ through its functional arginine-glycine-aspartic acid (RGD) cell binding sequence (Thalmann et al., 1999; Desai et al., 2007). Prostate cancer growth and progression is shown to be mediated by paracrine and autocrine signaling of OPN (Thalmann et al., 1999). CD44-OPN interaction induces cell migration out of the bloodstream to sites of inflammation. The migration of cells and subsequent invasion at distant sites involves a complex sequence of events (Weber et al., 1996). Variant CD44 isoforms bind to OPN independent of RGD sequences present at the N-terminal domain of CD44 (Katagiri et al., 1999). OPN binding to CD44 variants/beta1-containing integrin promotes cell spreading, motility, and chemotactic behavior in rat pancreatic carcinoma (Katagiri et al., 1999). OPN increases surface expression of standard CD44 (sCD44) in osteoclasts and both sCD44 and variant isoforms in human melanoma and PC3 cells (Chellaiah et al., 2003; Samanna et al., 2006; Desai et al., 2007). Osteopontin regulation of surface expression of CD44v6 and sCD44 was observed in breast and hepatocellular cancer cells (Gao et al., 2003; Khan et al., 2005).

### Matrix metalloproteases (MMPs)

Matrix metalloproteinases (MMPs) are important extracellular matrix proteins that are involved in degradation of the extracellular matrix. They are also important during development, wound healing, bone resorption, and angiogenesis (Paiva and Granjeiro, 2014). There is evidence suggesting that MMP-9 and CD44 associate in mouse and human tumor cells resulting in MMP9 activity localization on the cell surface (Yu and Stamenkovic, 1999; Gupta et al., 2013a). The interaction of CD44 and proteolytic form of MMP-9 is particularly involved in the invasion of prostate cancer cells (PC3) derived from bone metastases (Desai et al., 2007). Therefore, the ability of CD44 to localize proteolytically active MMP-9 to the tumor cell surface is important for tumor invasion (Yu and Stamenkovic, 1999).

## CD44 role in migration/invasion, angiogenesis, and bone metastasis

### Migration and invasion

CD44 receptor has the potential to integrate adhesive and signaling activities to modulate migration/invasion processes during cancer progression (Lokeshwar et al., 1995). The mechanisms by which CD44 receptors mediate migration, proliferation, survival of tumor cells through HA-mediated signaling have been widely studied (Bourguignon et al., 1998, 2001, 2004; Kuniyasu et al., 2001; Wang and Bourguignon, 2006a,b; Wang et al., 2009). Changes in cell shape and formation of adhesive structures are regulated by the dynamic regulation of the actin cytoskeleton. The dynamic regulation of the actin cytoskeleton and the specialized structures involved in migration are regulated by the temporal and spatial localization of actin-binding proteins (Chellaiah et al., 2000; Linder and Aepfelbacher, 2003; Desai et al., 2008). The surface expression of CD44 along with its interaction with matrix metalloproteinase 9 (MMP9) on the surface of the cell results in secretion of active MMP9, migration, and invasion of PC3 cells (Desai et al., 2007, 2008; Gupta et al., 2013a). Disruption of CD44/MMP9 interaction on the cell surface reduces migration and invasion of PC3 cells. When MMP9 is knockdown, CD44 expression switches to variant 6 (v6) isoform. This results in a less invasive phenotype due to lack of expression of sCD44 and inability to form invadopodia (Gupta et al., 2013a). CD44v6 expression inversely correlates with pathologic stage and disease progression and positively correlates with PSA-free survival in prostate cancer (Ekici et al., 2002). However, expression of CD44v6 in non-metastatic rat carcinoma cells has been shown to convert them into metastatic cells and promote tumor progression (Günthert et al., 1991; Seiter et al., 1993). Furthermore, CD44v3 has been shown to upregulate the function of cytoskeleton through ankyrin to activate the actomyosin contractile complex in order to mediate cell migration in head and neck squamous carcinoma cell line. Transfection of v3 cDNA into non-expressing cell lines also resulted in a significant increase in cell migration but not proliferation (Franzmann et al., 2001; Wang et al., 2007). CD44 variants have also been shown to function as a co-receptor for the activation of growth-promoting tumor receptor tyrosine kinases (Orian-Rousseau et al., 2002, 2007).

### Angiogenesis

The formation of new blood vessels (angiogenesis) is required for tumor cell to disseminate and migrate to distant organs. Past studies have identified CD44 expression on endothelial cells (Liesveld et al., 1994; Xu et al., 1994) and this controls the formation of blood vessels (Trochon et al., 1996; Savani et al., 2001). Inhibition of CD44 therefore results in impaired formation of vessel-like networks (Savani et al., 2001; Cao et al., 2006). Endothelial cells were found in increased numbers in prostate cancer tissues in relation to normal tissues (Wang et al., 2013). When CD44-null mice was used to study *in vivo* angiogenic responses, wound healing and vascularization were both impaired in matrigel implants. Therefore, metastasis formation is also linked to vascular CD44 expression (Cao et al., 2006). Adhesion of cancer cells to vasculature and enhanced expression of CD44 (CD44s and/or CD44v) by angiogenic factors (e.g., VEGF) produced by tumor cells might lead to facilitated extravasation via angiogenesis. Furthermore, the role of CD44 in tumor angiogenesis is enhanced by its binding to immobilized HA (Griffioen et al., 1997). CD44 variants are shown to have binding domains for various growth factors including vascular endothelial growth factor (VEGF), heparin-binding basic fibroblast growth factor and heparin binding epidermal growth factor (Bourguignon et al., 1998, 1999; Kalish et al., 1999). Analysis of tissue microarray and lysates of prostatic tumor cells showed that OPN and VEGF expression was more pronounced in prostate cancer as compared to benign or normal prostate tissues. It was suggested that an increase in micro vessel number and expression of CD44 might be useful diagnostic markers of metastasis of breast cancer (Ozer et al., 1997).

### Bone metastasis

Breast and prostate cancer cells ability to metastasize to bone is based on their ability to arrest on, adhere to, and extravasate across the bone marrow endothelium into the underlying bone matrix (Draffin et al., 2003). In prostate cancer cells, the selective adhesion of these cells to bone marrow epithelium is based on the role of adhesive properties of integrin receptors. Prostate cancer cells have been involved in strong interaction with the bone marrow endothelial cells (Draffin et al., 2004). There currently exists a dissension between clinical and experimental data in literature regarding the importance of sCD44 in breast cancer disease progression. A recent study suggests that breast cancer models show the expression of CD44 standard and variant isoforms which increase disease-progressing and metastatic behavior (McFarlane et al., 2015). HA and CD44 co-localize in the bone marrow sinusoidal epithelium, which is a site of metastasis of breast cancer. This suggests the contribution of HA-CD44 to the efficiency of distant metastasis to bone in breast cancer cells (McFarlane et al., 2015). Cells producing low levels of CD44 have lower ability to form tumor sphere *in vitro*. Furthermore, CD44 is a marker for cancer stem cells (Jaggupilli and Elkord, 2012; Cho et al., 2015; Stivarou and Patsavoudi, 2015) and CD44 expressing cancer stem cells increases the likelihood of bone metastases through its interaction with HA. Therefore, CD44-HA interaction could be a potential target for reducing bone metastases. CD44 signaling in prostate cancer cells has also been shown to regulate key proteins (i.e., RANKL and MMP9) involved in osteoclast differentiation and tumor metastasis (Gupta et al., 2012). Runx2 is a master transcription factor with important roles in osteoblast differentiation. Transcription of many osteoblast and bone formation related factors such as OPN, osteocalcin, and collagen type I are regulated by Runx2 (Akech et al., 2010).

## Role of CD44 as a transcriptional factor

Proteolytic cleavage that occurs at the extracellular domain releasing soluble CD44 has long been recognized. However, recent studies have shown that CD44 can undergo further sequential proteolytic processing by membrane type 1 matrix metalloproteases (MT1-MMP) and presenilin-1/y-secretase to produce the extracellular domain and intracellular domain (ICD) fragments. Presenilin-1/y-secretase cleavage occurs at the intramembrane site releasing two cleavage products of ~25 and ~16 kDa size. The 12 kDa ICD translocates to the nucleus to activate transcription of several proteins including CD44 itself (Okamoto et al., 2001; Nagano and Saya, 2004; Thorne et al., 2004). Consequently, if this cleavage can be inhibited through metalloprotease inhibitors, it can serve as a therapeutic way of preventing tumor progression and metastasis (Nagano and Saya, 2004). The translocation of CD44-ICD to the nucleus initiates the process of transcriptional regulation via it binding to novel promoter response element thereby regulating transcription of several genes that are involved in cell survival during stress, inflammation, oxidative glycolysis, tumor invasion (Okamoto et al., 2001; Miletti-González et al., 2012). This suggests a mechanism for the multifunctional role of CD44 in cancer cell metastasis and metabolism (Miletti-González et al., 2012). Nuclear translocation of the intracellular domain also shown to interact with stemness factors (Cho et al., 2015). CD44-ICD is linked with the regulation of MMP-9 gene in prostate and breast cancer cells through its interaction with the transcriptional factor RUNX2 (Miletti-González et al., 2012).

## Conclusions

The multifunctional glycoprotein CD44 can undergo alternative splicing events to produce CD44 variant isoforms that are more restricted in their distribution as compared to the standard CD44 isoforms (Rall and Rustgi, 1995). The ubiquitously expressed cell surface protein is primarily involved in aggregation, migration, and activation of cells, these functions are mediated through the adhesive properties of CD44 (Heider et al., 1993). Initially described for hematopoietic stem cells, it has since been confirmed as a marker of cancer stem cells (Bourguignon et al., 1998). CD44 interacts with a variety of ligands and can undergo sequential proteolytic processing resulting in the generation of CD44-ICD. CD44-ICD is known to translocate into the nucleus to activate gene transcription (Okamoto et al., 1999). Though the information provided here provides a comprehensive review of the literature thus far, there exists some discourse in the effect of ICD as the main modulator of metastatic events in cancers. To further substantiate CD44's effect in metastasis, research into the specifics will need to be completed to address this.

## Author contributions

LS and MC drafted the manuscript and equally contributed in editing and rewriting final contents.

## Funding

This work was supported by a research grant to MC from the National Institute of Health - National Institute of Arthritis and Musculoskeletal and Skin Diseases (5R01AR066044).

### Conflict of interest statement

The authors declare that the research was conducted in the absence of any commercial or financial relationships that could be construed as a potential conflict of interest.
